# Atorvastatin Confers Renoprotection and Modulates Inflammation in Diabetic Rats on a High-Fat Diet

**DOI:** 10.3390/life15081184

**Published:** 2025-07-25

**Authors:** Minela Aida Maranduca, Andreea Clim, Daniela Maria Tanase, Cristian Tudor Cozma, Mariana Floria, Ioana Adelina Clim, Dragomir Nicolae Serban, Ionela Lacramioara Serban

**Affiliations:** 1 Department of Morpho-Functional Sciences II, Discipline of Physiology, “Grigore T. Popa” University of Medicine and Pharmacy, 700115 Iași, Romania; minela.maranduca@umfiasi.ro (M.A.M.); clim.andreea@umfiasi.ro (A.C.); dragomir.serban@umfiasi.ro (D.N.S.); ionela.serban@umfiasi.ro (I.L.S.); 2 Internal Medicine Clinic, “St Spiridon” County Clinical Emergency Hospital, 700111 Iași, Romania; floria.mariana@umfiasi.ro; 3 Department of Internal Medicine, “Grigore T. Popa” University of Medicine and Pharmacy, 700115 Iași, Romania; 4 “George I.M. Georgescu” Institute of Cardiovascular Diseases, 700503 Iași, Romania; 5 Doctoral School of Medicine, “Victor Babes” University of Medicine and Pharmacy Timisoara, Eftimie Murgu Square 2, 300041 Timisoara, Romania; adelina.clim@umft.ro

**Keywords:** statin, inflammation, diabetes mellitus, obesity, pleiotropy, renoprotective

## Abstract

Objective: Uncovering the renoprotective and anti-inflammatory effects of atorvastatin treatment in diabetic-and-obese rats by employing traditional renal function indicators (urea and creatinine) and four prototypical cytokines (IL-1β, il-6, IL-17α, TNFα). Method: Twenty-eight male Wistar rats, aged 6 months, 350–400 g, were randomized into four groups. The first group, G-I, the denominated control, were fed standard chow over the whole course of the experiments. The rodents in G-II were exposed to a High-Fat Diet. The last two groups were exposed to Streptozotocin peritoneal injection (35 mg/kg of body weight). A short biochemical assessment was performed before diabetes model induction to ensure appropriate glucose metabolism before experiments. Following model induction, only rodents in group G-IV were gradually introduced to the same High-Fat Diet as received by G-II. Model confirmation 10 days after injections marked the start of statin treatment in group G-IV, by daily gavage of atorvastatin 20 mg/kg of body weight/day for 21 days. At the end of the experiments, the biochemical profile of interest comprised typical renal retention byproducts (urea and creatinine) and the inflammatory profile described using plasma levels of TNFα, IL-17α, IL-6, and IL-1β. Results: Treatment with Atorvastatin was associated with a statistically significant improvement in renal function in G-IV compared to untreated diabetic rodents in G-III. Changes in inflammatory activity showed partial association with statin therapy, TNFα and IL-17α mirroring the trend in urea and creatinine values. Conclusions: Our results indicate that atorvastatin treatment yields a myriad of pleiotropic activities, among which renal protection was clearly demonstrated in this model of diabetic-and-obese rodents. The statin impact on inflammation regulation may not be as clear-cut, but the potential synergy of renal function preservation and partial tapering of inflammatory activity requires further research in severely metabolically challenged models.

## 1. Introduction

A critical building block in the physiopathology of most chronic diseases, inflammation consists of a complex intertwined molecular network with two virtually independent angles: (1) a maintenance arm, which acts asymptomatically for maintaining the individual homeostasis around the normal ubiquitous stressors; (2) a recovery arm with clinically discernable rectifying effects. Should the latter arm act for an exceedingly long period after the ceasing of the disturbing stimulus, the rectifying component of inflammation gains pathological significance [1]. In these instances, chronic low-grade inflammation (LGCI) is perpetuated by the synergism between increased Nuclear Factor NF-κB (NF-κB) translocation and reduced inhibition by Adenosine Monophosphate Kinase (AMPK). Reduced anti-inflammatory activity hails the departure from homeostasis through pathological prolongation of the recovery phase of the inflammatory response. This deficiency in negative feedback within the inflammatory cascade enhances the LGCI state [2].

This new concept justifies the presence of a prominent basal inflammatory profile in two prototypical disorders: obesity and diabetes mellitus [3,4,5]. The dysregulation of multiple metabolic pathways suitably characterizes obesity from a physiological standpoint. Adipose cell necrosis and necroptosis constitute a powerful attractant for macrophages, which in turn locally amplify the inflammasome activity. Enhanced nuclear translocation of NF-κB is ultimately responsible for conversion to and maintenance of a catabolic metabolism. Tumoral necrosis factor α (TNFα) and IL-1β have been proven as mediators for LGCI in obesity, since targeted antibody neutralization improves metabolic balance [5,6]. Likewise, diabetes mellitus is characterized by cytokine profile dysregulation, among which IL-1β, IL-6, IL-18, and TNF-α are the most representative. Upregulation of NF-κB translocation in endothelial cells and hepatocytes, accompanied by tissular inflammatory damage in the same sites, is the most likely mechanism for LGCI [7,8].

Low-grade chronic inflammation is a double-edged sword. On one hand, the persistent pro-inflammatory imbalance impairs the normal defense function of the immune system, potentially increasing the damaging effect of otherwise trivial stressors. On the other hand, a delayed or insufficient anti-inflammatory response triggers several metabolic dysregulations, enclosing a vicious self-fueled circle of uncontrolled inflammation. Kidneys are dragged into this self-destructive cycle, where oxidative stress contributes to a decline in glomerular function, which in turn amplifies the damaging potential of uremic retention products [9]. Remarkably, recent research has advocated for NF-κB as the master regulator of renal fibrosis, the principal mechanism of renal decline progression [10,11].

Cardiovascular disease is another direct consequence of perpetual inflammation, the atherosclerotic plaque representing the pivotal point [12]. Since the advancement of the “Cholesterol hypothesis” in the development of cardiovascular disease, several lipid-lowering drug classes have been pushed to more consistent clinical use, regardless of the lipid status [13]. The anti-inflammatory benefits of statins were suspected more than 25 years ago and are now canonically included under the umbrella of pleiotropic effects [14,15]. Newer proposed mechanisms revolve around inflammasome activity, with particular focus on pro-inflammatory cytokines (IL-1β, IL-6, TNFα), but evidence is still disputed [16,17,18].

In order to delineate some of the pleiotropic benefits of statin therapy, we compared the anti-inflammatory and renoprotective effects of Atorvastatin in a diabetic model of rats fed with a high-fat-content diet against similar metabolically challenged rodents—either obese or diabetic. Between groups, we compared traditional markers of renal function and levels of representative cytokines. Lastly, we checked if and to what extent the renal function at the end of the experiment correlated to the inflammatory profile.

## 2. Materials and Methods

### 2.1. Animals

Twenty-eight (28) male Wistar rats, aged 6 months (350–400 g), were procured from the Cantacuzino Institute, Bucharest, Romania, as a part of a complex experimental study. The animals were housed in CEMEX at Grigore T. Popa University of Medicine and Pharmacy, Iași, Romania, and conditioned at 20 ± 2 °C, 50 ± 5% humidity on a 12 h light/12 h dark cycle. Animals had individually ventilated cages and free access to water and assigned food. After 14 days of acclimatization, the rats were initiated in the protocols.

### 2.2. Ethical Approach

The experimental investigation was according to the European Directive 2010/63/EU. Approval was obtained from the ethics committee of the university (No. 277/26.02.2023) and also from the Romanian National Sanitary Veterinary and Food Safety Authority (No. 61/27.04.2023).

### 2.3. Experimental Design and Treatment Groups

To evaluate biochemical changes elicited by Atorvastatin treatment on inflammation and renal function, rats were grouped into four equal-count groups (*n* = 7). All animals were exposed to 14 days of an acclimatization period to reduce the level of perceived stress. During this phase, the subjects were provided with a standard chow diet and had unrestricted access to water. On day 15, 7 rats were assigned to each of the 4 groups comprising the current experiment (see Table 1).

Between days 15 and 18, the animals in groups II and IV were gradually exposed to the new diet. Introduction of the high-fat-content diet (HFD) with 2% cholesterol—prepared by the Cantacuzino Institute, Bucharest, Romania—was accomplished by incrementally mixing the two feeds over the course of 4 days. We aimed at minimizing the impact of the presumably different appetites of the animals on the newly introduced chow. The macronutrient composition of the employed diets is found in Table 2.

Atorvastatin (Sortis, Pfizer, McPherson, KS, USA, No NM4027) was mixed with distilled water and administered once a day orally, by gavage, using a sterile dispositive for every rat (16 G × 1.1/2, Popper and Sons, New Hyde Park, NY, USA). The solution was administered only to animals in the group G-IV, after confirmation of the experimental model of diabetes. All other animals received distillate water using the same administration procedure to account for manipulation stress and any inconsistent low-grade trauma determined by the gavage procedure. An extensive blood-derived biochemical evaluation was performed on days 14 (beginning of the study) and 50 (end of experiments, animals were sacrificed).

Gross Necropsy: on the 50th day after laboratory habitation (i.e., 36 days from initiation of the experimental protocol), tissue and blood sampling were performed on all animals. After a 12 h fast, deep anesthesia using an intraperitoneal injection of ketamine-xylazine (MilliporeSigma, Taufkirchen, Germany), dosed 50–5 mg/kg body weight, was closely followed by euthanasia through rapid decapitation. This choice aligns with the literature on euthanasia-generated errors in blood-derived parameters [19,20,21,22]. Blood sampling for biochemical analysis at the end of the experiment was performed by cardiac puncture. From each rodent, 4 mL of blood specimen was collected in special vacutainer tubes with a clot activator solution designed for biochemical assays. Postmortem examination included a rigorous evaluation of bodies and internal cavities, and systematic tissue sampling.

### 2.4. Type 2 Diabetes Mellitus Rodent Model Induction and Confirmation

Our experiment employed a streptozotocin (STZ)-induced type 2 Diabetes Mellitus (T2DM) rat model. In accordance with existing studies concerning the reliability of this model (induction and survival rates), we adjusted the dose of STZ (MilliporeSigma, Taufkirchen, Germany) to 35 mg/kg of body weight. Rats were weighed, and a minimally traumatic, basal assessment of glycemia was required to ensure that the specimens presented normal blood glucose regulation prior to streptozotocin injection. STZ solution was prepared to a concentration of 3.5 mg per mL by dissolving the powder form of STZ in the appropriate volume of 0.1 M sodium citrate buffer at pH = 4.5 (MilliporeSigma, Taufkirchen, Germany) using an ultrasonic warm bath. After confirming normal basal glycemia, the 14 rats belonging to groups III (diabetic only) and IV (diabetic, obese, statin treated) were moved to a separate enclosure and each injected with a single intraperitoneal dose of STZ, volume depending on weight (e.g., a 450 g rat requires 4.5 mL solution).

Immediately after this procedure, the rodents were returned to their cages and provided free access to assigned chow and a 10% glucose water solution, anticipating the dangerous hypoglycemia that may manifest within the first 24 h after the injection. The glucose-rich solution was then withdrawn. The animals had ad libitum access to water and the assigned diet. The rats were checked for model development at 7 and 10 days following the injection (i.e., days 25 and 28 counted from the rodents’ arrival in the laboratory setting) by minimally invasive measurement of glycemic values. Confirmation of the model was accepted in those rats that returned a blood glucose concentration of 200 mg/dL or more on the 10th day. All 14 rodents (100%) exposed to the induction procedure survived and developed T2DM.

### 2.5. The Complete Biochemical Assay Protocol

Less than 30 min following blood drawing, the vacutainer tubes were centrifuged at 1500× *g* for 15 min at 4 °C. Measured parameters included traditional renal function parameters (urea, creatinine), cytokines characterizing systemic inflammation (IL-1β, IL-6, IL-17α, TNFα), C-reactive protein, lipid profile (Total Cholesterol, TC; High-density lipoprotein Cholesterol, HDL-C; Low-density lipoprotein Cholesterol, LDL-C; Triglycerides, TG), and glycemia. The automated analyzer ACCENT-S120 (PZ Cormay, Warsaw, Poland) was used to determine the results for biochemistry by adhering to manufacturer protocols. All reagents required for the traditional biochemical parameters (i.e., except the 4 cytokines) were procured from Cormay Diagnostics (PZ Cormay, Warsaw, Poland). For the inflammatory profile, we ordered a specifically designed Rat Multiplex ELISA Panel, consisting of 4 cytokines (IL-1β, IL-6, IL-17α/CTLA-8, TNFα). We used the prespecified wash and buffer solutions from AssayGenie (Dublin, Ireland, UK) and followed the instructions received along with the reagents. The CytoFLEX Flow Cytometer (Beckman Coulter, Life Sciences, Indianapolis, IN, USA) Violet–Blue–Red series was the machine employed for quantitative signal detection for cytokine profiles.

### 2.6. Additional Glycemia Assays in Diabetic Groups

Aside from the complex biochemical analysis on day 14 and day 50, groups III and IV (the diabetic specimens) were exposed to more frequent measuring of the blood glucose value. Three additional glycemia measurements were performed using a Code-Free glucometer: prior to diabetes induction and at 7 and 10 days following STZ injection. Each animal was moved to a separate room, held in a restrainer, and its tail was massaged to bring sufficient blood to the tip. After cleaning with a 96% alcohol solution, the tip of the tail was punctured using a sterile needle for each specimen. The drop of blood was collected using a strip and inserted into a glucometer (STANDARD CodeFree PlusR, SD BIOSENSOR Healthcare, Haryana, India).

### 2.7. Statistical Analysis

Visualization for each biomarker of interest was performed using box plots, delineating the first and third quartiles, interquartile range (IQR = Q3 − Q1), and the median and average of the sample. We chose to represent Q1 and Q3 by excluding the median value in each group of 7 specimens. Values were represented as outliers if they exceeded the box plot whiskers situated 1.5 IQR apart from respective quartiles. Normality was assessed by the Shapiro–Wilk test and the symmetry of the box plot in samples with less than 10 animals, while in larger samples, by performing Q-Q plots and qualitatively deciding the resemblance of a parametric distribution.

In situations where distributions were parametrical (normal), we preferred Student’s *t*-test, a tool powerful even with small sample sizes. Non-parametrical distributions required bootstrapping tests—which is, by design, more robust to non-normality and a small sample size—to characterize the difference in means between groups. Lastly, correlation/dependence analysis was performed between renal function indicators and cytokines. In the situation where the distribution of both the dependent and independent variables was approximately normal, we performed correlation analysis via the Pearson test, at a statistically significant cutoff value of α = 0.05. Non-normal distribution of either the dependent or independent variable demanded the use of Kendall’s Tau dependency factor to preserve most of the original character of data. Data analysis was carried out using IBM SPSS Statistics for Windows, Version 25 (SPSS Inc., Chicago, IL, USA). Box plots were generated using the Seaborn library 0.12.2 version.

The sample size was calculated in the vision of creatinine concentration reduction. We used data from our previous work and from the literature for the effect of statin treatment, an expected difference of at least 0.20 mg/dL under the statin dose and duration employed in our study. Previous unpublished data using similar diabetic-and-obese rodents yielded a relatively uniform distribution of creatinine values across all study groups, the highest SD being 0.12 mg/dL. At 80% power and 5% type 1 (alpha) error, the minimum sample size is 6 rats. We decided to use 7 specimens in each group to account for unexpected death.

### 2.8. Statistical Tests Were Chosen to Retain Most of the Original Data Information

The skewness (or proximity to normality) of the distributions dictates the test to be used for proper comparison. The choice to use the bootstrapping technique in non-parametrical distribution comparisons was supported by the higher confidence returned by this test, in comparison to the Mann–Whitney U test (Wilcoxon rank test). Rank-based tests (Mann–Whitney U, Spearman correlation coefficient test) lose an important part of the initial information through ranking, while bootstrapping maintains the original information, scale, and unit of measure. This is especially important for the interpretation of values erratically spread over a broad range.

Distributions of each of the four cytokines in the whole study population of 28 animals showed a bimodal appearance, with a clear tendency of very low values for the control group, distinctly separated by the much higher values in the 21 animals exposed to human-induced metabolic disturbances. Creatinine and urea values exhibited a much narrower relative range and displayed normal distribution for both the 28 (whole) and 21 (experiment only) samples. Concerning correlation versus dependence analysis between renal filtration function indicators and cytokines, the choice was again dictated by the distribution normality of the two input variables. Parametrical curves allowed performing the Pearson test. Non-normal distributions were more accurately assessed from a dependence perspective by the Kendall Tau coefficient.

## 3. Results

**Figure 1:**
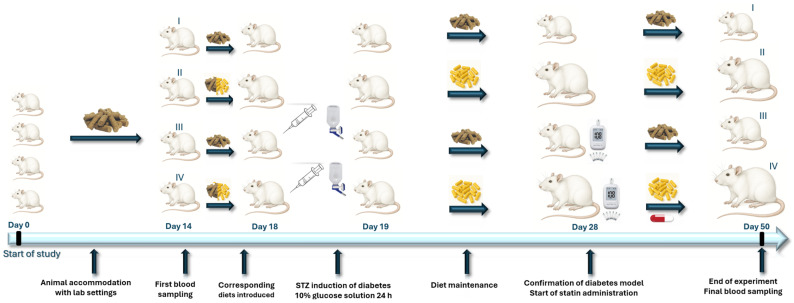
Experimental design. After 14 days of acclimatization, the 28 rats were allocated into 4 groups: group I (control group fed standard diet), group II (obese group, receiving high-fat-content diet (HFD) with 2% added cholesterol), group III (Streptozotocin-induced diabetic model fed with standard chow), and group IV (Streptozotocin-induced diabetic model fed with HFD). On day 18, all groups were receiving 100% of the assigned diet. Rodents from groups III and IV were induced diabetes by single intraperitoneal Streptozotocin injection, 35 mg/kg body weight, on day 18. Confirmation of the model was performed on day 28. Starting on day 29, animals in group IV received Atorvastatin 20 mg/kg body weight, orally, by gavage, every 24 h for the next 21 days. In all four groups, blood sampling was performed from the left retro-orbital plexus on day 14 and by cardiac puncture at the end of the experiment, on day 50.

Figure 1 briefly describes the experimental design, the essential details of the four independent groups, and the important moments over the course of the study when samples were collected for model confirmation and for analysis of the treatment effect.

**Figure 2:**
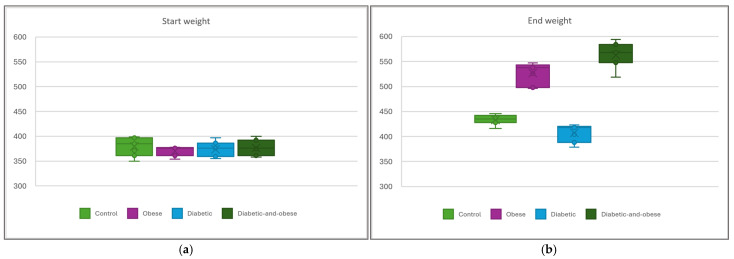
(**a**) On the left, the starting weights of the rats measured on day 1, corresponding to the four groups. (**b**) On the right, the corresponding weights on day 49.

Raw data for the results presented in this paper can be found in the Supplementary File, Table S1. Average starting and ending weights are provided, essential in assessing the impact of the diabetes model, disease severity, and the interaction with each diet (Figure 2a,b). While starting weights show a satisfactory uniformity of the laboratory animal, end weights highlight the role played by diet in the weight gain of the diabetic specimens. The diabetic animals in group G-III show a modest weight gain, lower than the control group G-I, typically expected in diabetic specimens fed with high-carbohydrate-content diets. The high-fat diet in group G-IV appears to rescue the growth curve, since these specimens exhibit the highest gain in the study.

**Figure 3:**
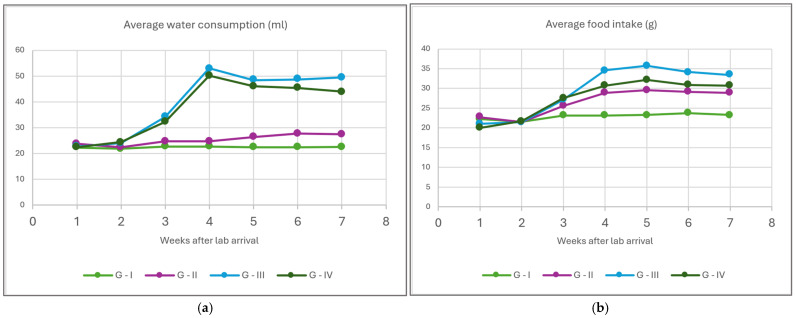
Weekly average water (**a**) and food consumption (**b**) in each study group.

Food and water intake values are provided as weekly means for easier interpretation (Figure 3a,b). Both parameters during the first two weeks are similar across the board for all groups, since this is still during the habitation period. The third week corresponds to the diet introduction and diabetes model induction in groups G-III and G-IV; therefore, some changes become apparent but not straightforward. From the 4th week onward, water consumption spikes in both diabetic groups in comparison to the control and obese groups, although the group fed with HFD appears to have marginally lower values than the diabetic rodents fed a normal diet. Food intake during the same period is highest in the diabetic group G-III, followed by the diabetic-and-obese group G-IV and then by the obese G-II group.

The statistical analysis was focused on the renal function parameters and cytokine profile, summarized in Table 3. If the distribution of a variable resembled a normal shape, values are described as Mean (Standard deviation). Otherwise, values are presented as Median (Q1, Q3).

At the initiation of the experiments, on day 14, some differences have already developed between groups. The mean glycemia value for group IV is statistically higher than the average glycemia in groups II and III. Similarly, the initial creatinine levels are higher in group G-III compared to G-I, the difference attaining statistical significance. Regardless, the absolute difference between groups does not question the model uniformity.

**Figure 4:**
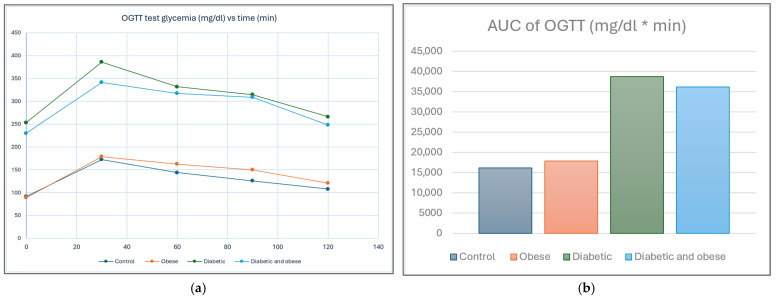
(**a**) Oral glucose tolerance test profile reported by time after solution administration (OGTT). (**b**) AUC for each group reported as mg/dL * min.

The oral glucose tolerance test (OGTT), alongside a-jeun glycemia values, was used for confirming successful diabetic model induction. In Figure 4a, we provide the glycemic profile as the mean values for each group with respect to the time elapsed since the administration of the oral glucose solution 20%. In Figure 4b, mean values, reported as the AUC for the OGTT test, for each group are reported.

Table 4 below presents histological proof for renal damage in the G-IV group suffering from a dual metabolic impairment, albeit treated with statin.

**Figure 5:**
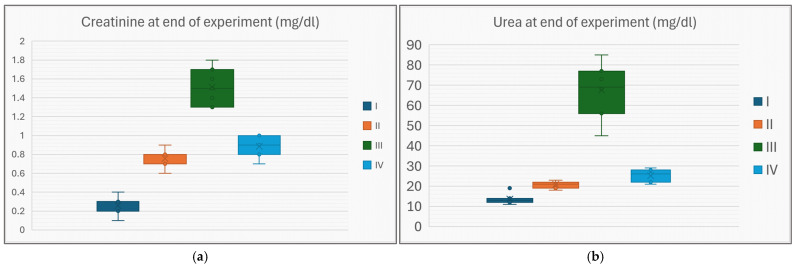
Creatinine (**a**) and urea (**b**) levels at the end of the experiment in each of the study groups.

**Figure 6:**
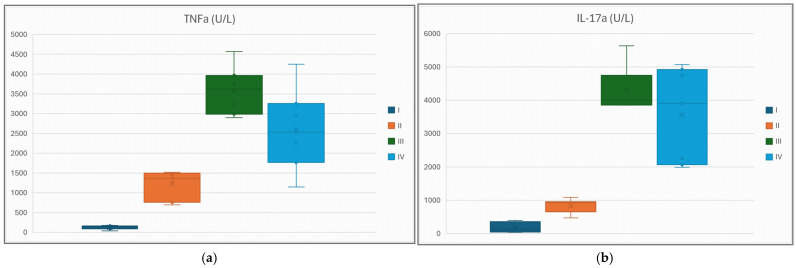
Plasma levels of TNFα (**a**) and IL-17α (**b**) at the end of the experiment in each study group.

Box plots for the traditional renal function markers (plasmatic creatinine and urea) exhibit closely matching behavior with respect to mean values and statistical significance (Figure 5a,b). In both markers, average values were ordered as G-I < * G-II < * G-IV < * G-III, the differences between each two consecutive groups being statistically significant (denoted by *). The next two figures (Figure 6a,b) are box plot representations of two key inflammatory cytokines. In both cases, the mean value in each group paralleled the trend previously displayed by urea and creatinine: G-I << G-II << G-IV << G-III. TNFα presents statistically significant differences between each two consecutive groups across the whole experiment population, while IL-17α preserves the statistical significance only for the first two mean differences (i.e., the average value of IL-17α for group IV is lower than for group III, but the *p* value = 0.095, >0.05).

The trend displayed so far by the previous indicators of renal function and inflammatory activity between all four groups does not extend to the other two interleukins. As depicted in Table 1, the values of IL-1β and IL-6 in group G-I control are tightly packed towards the very low end of the range. Group IV, however, presents the highest mean value for both IL-1β and IL-6. There is a statistically significant difference for the former cytokine between group IV and group III, with the mean values ordered as G-I << * G-III < * G-II < G-IV. Lastly, the trend in average values for IL-6 could be ordered as G-I << * G-II < * G-III < G-IV, where * denotes statistical significance for the mean difference.

**Figure 7:**
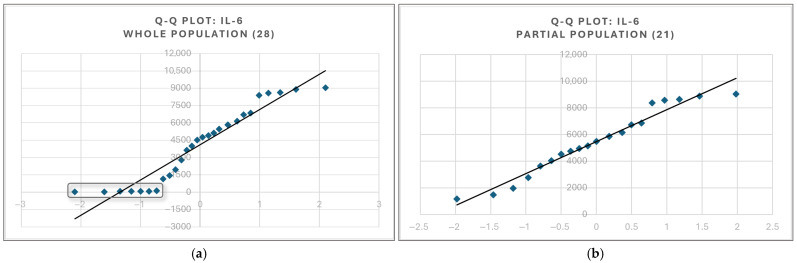
Q-Q plot for IL-6 values displaying a bimodal distribution of values; first 7 values pertaining to group G-I control (**a**; left, enclosed) are distinct from the 21 entries belonging to the other groups (**b**).

The closely matched box plot distributions between the two renal filtration markers and TNFα and IL-17α prompted further investigation to find whether there is any significant correlation between individual indicators of renal function and markers of inflammatory activity. On the whole experiment population size (all 28 rats), the distribution of none of the four mentioned biochemical variables exhibited a parametrical curve. The distributions of all cytokines retrieved a bimodal aspect of the quartile–quartile (Q-Q) plot, with clear delineation between the first 7 values, corresponding to rodents in control G-I, and the rest of the 21 values, belonging to G-II to G-IV. (Figure 7a,b). The plot for IL-1β is representative of all cytokines measured in our study.

The non-parametrical distribution of the data for the whole population size of 28 rodents required any degree of dependence between (1) any of the four cytokines and (2) creatinine or urea to be assessed using the Kendall Tau test to maintain the highest confidence concerning the original data. The values and statistical significance are summarized in Table 5. Separating the control group from the entire population yielded 21 values that resembled a parametrical distribution for each of the four cytokines (distribution in Figure 7b representative). This allowed for correlation hypothesis testing using the Pearson test, with the resulting values displayed in Table 5.

## 4. Discussion

### 4.1. Obesity and Diabetes Individually Impair Renal Function

The histological extent of lesions suggests a definite inflammatory process in the renal matrix and glomeruli, but the extent of the disruptive process as resulting from an average nephron pool loss is limited. This is consistent with the limited duration of our experiment, with definite development of chronic kidney disease taking sometimes as long as 24 weeks. However, expecting this limited extent of damage, we chose a relatively short—proportionate—duration of statin treatment. Our results demonstrate a statistically significant higher retention of creatinine and urea in plasma—a reflection of the respective reduction in renal function—in every single experimental group (G-II to G-IV) in comparison to the control group G-I. The diabetic group G-III demonstrates the highest values for both creatinine and urea across the whole experiment population. The plasma concentrations of retention byproducts in both G-II and G-III are statistically significantly higher compared to the control G-I, indicating that each condition independently leads to important impairment in kidney function. The assumption of concurring actions in a model exhibiting obesity and diabetes simultaneously should translate into outcomes—renal and inflammatory—with values skewed from normality, the dimension of departure from the normal range at least as big as in any of the models exhibiting an individual condition.

A valuable fraction of glomerular pool has to be lost before either creatinine or urea serum concentrations exceed the normal range [23]. Consequently, blood urea nitrogen—an indicator derived from the blood urea concentration—remains within the accepted normal range until moderate-to-advanced kidney disease. Plasmatic urea exhibits a steeper increase, with the glomerular filtration rate approaching values characteristic of end-stage renal disease, unlike the linear increase characterizing blood creatinine levels [24,25]. The plasma creatinine concentration functions as an inverse surrogate indicator for renal function at any given time. In our study, the box plots for all three experimental groups G-II to G-IV show creatinine values above 0.5 mg/dL, reflecting important renal damage at the end of the study.

Owing to the systemic endocrine and autonomic disruptions, obesity severely impacts renal health. The direct mechanical compression by surrounding renal capsule fat, increased activity of the Renin–Angiotensin–Aldosterone axis, and increased brain-mediated sympathetic activity on the large-scale hemodynamic level all contribute to an initial phase of glomerular hyperfiltration. Further cemented by LGCI and altered lipid metabolism, the degradation of renal function becomes unavoidable and progressive unless the trigger stimulus—obesity—is removed [26,27]. In humans, diabetic kidney disease accounts for almost 50% of chronic kidney disease (CKD), primarily driven by local aberrant cellular metabolism, increased tissular damage propensity, and exaggerated inflammatory infiltrate [28]. All these mechanisms culminate with phenotype transition to myofibroblasts, which prime local fibrosis and nephron loss [29]. The deleterious effects mediated by diabetes and obesity upon renal function are showcased by human studies that prove the beneficial impact of weight loss upon renal function and metabolic homeostasis, irrespective of the type of DM [30,31].

### 4.2. Atorvastatin Rescues the Renal Function in the Diabetic-and-Obese Rat

Our research shows that in this instance of the diabetic-and-obese rat model, Atorvastatin was effective in reducing both creatinine and urea to levels lower than the ones displayed by the solely diabetic rodent. These differences were statistically significant in favor of the statin-treated group, highlighting the treatment effectiveness in spite of HFD administration. The two retention products showed average plasma concentrations statistically significantly higher in group G-IV compared to the obese group G-II. This suggests that in an obese-and-diabetic rodent model, statin was not as efficient canceling out the renal impairment induced by diabetes in comparison to the solely obese rodent. Had statin treatment been effective in completely reversing the renal damage induced by hyperglycemia in the obese specimens, the renal function of rats in G-IV would have been closer to the values obtained in the obese group G-II; this was not the case.

Statins are currently recommended for the primary prevention of cardiovascular disease, with the guidelines advocating for more than 10% reduction in a 10-year risk [32]. The level of evidence is moderately strong (B) for adults aged between 40 and 75 and with at least one cardiovascular risk factor (e.g., dyslipidemia, diabetes). Basic science provides satisfactory results after statin administration in several models of rats fed with a high-fat-content diet or cholesterol-rich diet. The benefits translate to improved histology (reduced mesangial proliferation, tubulointerstitial fibrosis, and less lipid droplet accumulation), as well as function (revealed by lower oxidative stress and inhibited expression of markers of renal injury) [33,34,35]. In clinical settings, the renoprotective effects are not as clear cut. There is conflicting evidence for a potential reduction in the yearly glomerular filtration rate decline, with statin occasionally exhibiting a disconcerting performance compared to non-statin lipid-lowering therapy [36,37]. Nevertheless, at advanced stages of renal function loss—starting from G3 CKD—statins consistently reduce the aging-dependent decline in glomerular function [38,39].

### 4.3. The Concentrations of Creatinine and Urea Are Reliably Correlated to IL-17α and TNFα

In order to assess the effect direction and size of the drug from a renal and inflammatory perspective, we analyzed the values for these parameters at the end of the experiment in comparison to values recorded prior to treatment administration. The trends exhibited by the average values of TNFα and IL-17α closely mirrored the trends in both renal retention products. The mean difference between groups G-IV and G-III attained statistical significance for both urea and creatinine, advancing the hypothesis that atorvastatin treatment is associated with an important rescue of the renal function in the dually impaired rodent model of diabetes and obesity. Remarkably, these effects are replicated by both TNFα and IL-17α levels at the end of the study. While the mean difference for TNFα between groups G-IV and G-III was statistically significant, this significance did not apply for IL-17α, in spite of a similar trend of means. Atorvastatin treatment is therefore associated with an important rescue of inflammatory cascade activity.

The inflammation cascade relies upon IL-17α for mediating some of its deleterious effects. Interleukin IL-17α is an important determinant of structural and functional renal decline in several rodent models of diabetic nephropathy and atherosclerosis [40]. This cytokine is the pivotal element in the balance of the couple T-helper 17/T-regulatory cells (Th17/Treg), the increase in IL-17α leaning towards reduced regulatory activity and uncontrolled fibrosis. Early plaque formation, stabilization, and overall systemic vascular inflammation all rely upon IL-17α signaling [41]. IL-17α also impairs protective mechanisms against hyperglycemia-mediated renal damage and indirectly contributes to the progression of diabetic nephropathy by Th17-mediated fibrosis [42]. The pivotal contribution of IL-17α in the progression of CKD is strengthened by the restoration of glomerular barrier impermeability and the attenuation of pro-fibrotic and pro-inflammatory genes after monoclonal antibody blockade of the cytokine in rodent models [43,44]. These findings are matched in human prospective cohorts, the levels of IL-17α following the rate of uremic toxin accumulation over the course of renal decline, regardless of its acute or chronic nature [45,46,47].

An important cytokine regulator of renal function, TNFα is both a mediator for inflammatory effects and a direct player on the nephron. The differential activity of the two isoforms of TNFα receptors (TNFR1 and TNFR2) lays the foundation for a bimodal pattern concerning renal function: with steeping values of the cytokine, the resulting effect shifts from natriuresis to the induction of hypertension [48]. Indeed, toxin-induced nephropathy in rodents is the result of TNFα-mediated fibrosis [49]. In humans, both obesity and diabetic nephropathy are recognized entities for increased circulating TNFα [50,51]. Studies consistently report a negative association of the cytokine levels with the glomerular filtration rate and serum creatinine in CKD [51]. Despite that, the TNFα concentration parallels the decrease in the renal filtration rate; this cytokine is more likely an indicator of rapid loss of function rather than an active gear inside the destructive mechanism [52].

### 4.4. Atorvastatin Improves the Inflammatory Profile of Diabetic-and-Obese Rats, but Appearances May Be Deceiving

Administration of statin in group G-IV was associated with lower TNFα values in comparison to the diabetic group G-III, the difference attaining statistical significance. This finding points to the effectiveness of statins in this diabetic model, as the drug demonstrated a significant anti-inflammatory benefit, despite the animals being fed a high-fat diet. In cardiovascular disease patients, a recent meta-analysis showed that statins, regardless of dose, resulted in a statistically significant reduction in TNFα concentrations [53]. This observation matches CKD patients, where statin treatment resulted in a similarly statistically significant improvement of the concentration of the same cytokine [54]. The most recent meta-analysis focused on patients suffering from any form of chronic disease and highlights the high efficacity of Atorvastatin among its class in reducing levels of TNFα [55]. It is worth mentioning that, apart from the scarcity of randomized controlled trials (RCTs) assessing this cytokine, the vast weight of this effect arose from a single study on patients on statin therapy for more than 4 months. These findings cannot be reliably extended to our paper, since our treatment period was much shorter.

Statin therapy consistently demonstrates an improvement in TNFα levels. Part of this beneficial effect is the endothelial cell increased resistance to activation by TNFα in the presence of an adequate statin concentration [56]. Furthermore, atorvastatin mediates a reduction in TNFα release itself from mast cells; but, this effect is inconsistent across the statin drug class [57]. Through modulation of the NO balance in the endothelial cell layer, statins reduce the release of pro-inflammatory interleukins such as IL-1β and TNFα while increasing the expression of modulating molecules such as IL-10 [58]. Moreover, statins reduce the expression of class II major histocompatibility complexes on antigen-presenting cells, resulting in the reduction of the pro-inflammatory cytokine release and T-cell phenotype shift from T helper 1 to T helper 2 [59,60].

In our research, statin treatment in group G-IV resulted in a tendency to lower IL-17α values, albeit lacking statistical significance. Both obesity and diabetes models separately demonstrated a statistically significant increase in levels of this cytokine concentration compared to the control G-I group. When comparing G-IV with statin treatment, the impact generated by a high-fat diet upon IL-17 levels must be acknowledged [61]. The metabolic impairment driven by the simultaneous presence of diabetes and a high-fat diet was limited and reversed by statin treatment, when comparing IL-17α in groups G-IV to G-III.

While we could not find any meta-analysis concerning the effect of statin treatment upon IL-17α levels, there is a definite association between higher IL-17 concentrations and the odds for developing ischemic heart disease, a pathology that typically benefits from statin therapy [62]. There is limited evidence concerning the benefit of statin administration in humans, but statins demonstrated a reduction in T-cell proliferation mediated by IL-17α, along with increased resistance of endothelial cells to the same cytokine signaling [61,63]. These steps translate to synergic anti-inflammatory activity at multiple sites, through a reduction in the endothelial surface exposure of cell adhesion molecules, downregulation of IL-6 and IL-23 release from monocytes, and negative regulation of the subset T-helper cells 17, the culprit in modern therapeutic approaches for atherosclerosis [64,65].

### 4.5. Paradoxical Effects of Statin upon IL-6 and IL-1β Cytokines Are Likely an Illusion

In our study, Atorvastatin did not exhibit any beneficial effect on IL-6 values, group G-IV returning by far the highest mean IL-6 concentration in plasma across the whole experiment. IL-6 did not demonstrate any statistically significant correlation with either creatinine or urea at the end of the experiment in any of the groups. However, from a whole-experiment population size, the dependence coefficient Kendall τ maintained statistical significance. This dependency relies upon three aspects: (1) we included the whole population of 28 rats; (2) the control group exhibited the lowest values of both IL-6 and renal retention products; (3) the trends exhibited by renal function indicators were not fundamentally different from the trend exhibited by IL-6. Since the Kendall coefficient is a measure of dependency, even in the situation of a not perfect resemblance of trends, the high discrepancy between the values in G-I (situated in the very low end of the range) and those in G-II to G-IV accounted for the paradoxical statistical significance. The statistical significance of Kendall τ in the dependence analysis of IL-6 versus renal retention markers does not imply any notable effect of Atorvastatin.

The apparent worsening of IL-6 and IL-1β levels is the consequence of the absence of a diabetic-and-obese but not statin-treated group. Since diabetes and obesity are both forms of metabolic disturbances, it is likely that their simultaneous presence in an untreated rodent model will inflict more extensive organ damage than any of the individual components. Therefore, the hypothetical untreated model, which in our pilot study demonstrated poor survivability, would display IL-6 and IL-1β mean levels higher than both the G-II and G-III groups. We cannot infer the effect size of statin treatment on the two previous cytokines; we can only assume that the magnitude of the effect was lower than in the case of TNFα and IL-17α.

The existing literature is somewhat conflicting with respect to the role of statins in controlling levels of IL-6, two recent meta-analyses discrediting any role of statins for improvement of the IL-6 concentration in either cardiovascular or renal disease patients [53,54]. However, the majority of the weight comprising the plots for cardiovascular disease patients arises from studies older than 13 years, which may indicate some concerns in risk of bias assessment. A newer meta-analysis restricted to RCTs on subjects suffering from any chronic disease showcases a different perspective, depending on the treatment duration [55]. Newer studies involving statin therapy over less than 4 months show an overall null effect on IL-6 concentrations, while statin treatment of longer than 4 months demonstrates a statistically significant improvement in the forementioned cytokine levels. Lastly, a more extensive RCTs meta-analysis—albeit older than the previously mentioned ones—highlights a statistically significant improvement in IL-6 in patients suffering from any metabolic-related disorder [66]. The diabetic subgroup of patients, the most similar entity with our rodent model in group G-IV, displays significant improvement upon IL-6 concentration with statin therapy. However, the effect on IL-1β is less impressive; only one study included an overall not statistically significant result.

Statins have a positive impact on the nitric oxide (NO) balance at the level of endothelial cells through the synergic action of reduced degradation and increased endothelial NO synthase activity [67]. This modulation in local oxidative stress translates into a reduction in redox-sensitive mediators such as NF-κB, the master regulator for inflammatory activity. Consequently, the expression of several adhesion molecules such as intercellular cell adhesion molecule-1 (ICAM-1) and platelet endothelial adhesion molecule-1 (PECAM-1) inhibits the initial phases of inflammatory infiltration in the vascular wall [68,69]. Furthermore, statins reduce the production of pro-inflammatory cytokine IL-6 from circulating monocytes [70].

Even though statins are widely recognized as anti-inflammatory agents, the effect upon IL-1β is not as uniform. There are several reports of statins increasing the release of IL-1β through the activation of the NOD-like receptor family, pyrin-domain-containing (NLRP) 3 inflammasome [71]. Most importantly, this activation requires the presence of a priming agent for NLRP3, such as bacterial lipopolysaccharide 1. Mediated by a statin-dependent reduction in protein isoprenylation, followed by abnormal sensitivity of the inflammatory signaling pathways, the response in macrophage IL-1β secretion is enhanced by high circulating levels of the same interleukin [72]. However, not all is lost, as Simvastatin demonstrated a beneficial effect in hyperlipidemic models. Priming of NLRP3 by cholesterol crystals results in a drop in IL-1β levels [73].

### 4.6. A Word of Caution: Indiscriminate Statin Treatment May Not Be the Solution

Undoubtedly, statins are powerful lipid profile regulators, as they collectively reduce total cholesterol, the LDL fraction of cholesterol, and triglycerides, regardless of a higher body mass index [74,75]. Still, caution is key in the administration of statins to obese patients. While fundamentally at risk for metabolic syndrome, there are concerns regarding de novo T2DM development in obese individuals. Statin mediates intracellular cholesterol synthesis inhibition, which causes a shift towards extracellular uptake with undesirable metabolic consequences: impaired β pancreatic cell function and insulin resistance [76,77,78]. The upper age safe limit of 75 is another important clinical concern, as there are several inconclusive reports that incriminate statin medication as a determinant of severe muscular pain [79].

### 4.7. Limitations

There are a few strength points that highlight the value of our research. First of all, we are among the few researchers who have studied the complex model of diabetes and obesity, focusing on renal and inflammatory function. Secondly, we have strong evidence regarding the association between very accessible statin treatment and the rescue of renal function. Lastly, the inconsistency noticed in the improvement of the inflammatory profile is a glimpse into the difficult network enclosed by inflammatory cytokines and opens future perspectives for designing more in-depth basic research protocols.

However, our paper presents certain limitations that warrant caution when interpreting the findings. The experimental design, namely, the involvement of a single diabetic model in a single species, limits extrapolating the results to human and clinical medicine. For ethical reasons, each group consisted of no more than seven animals, allowing for reasonable statistical power and a low rate of error stemming from the small sample.

The fixed short duration of statin administration (21 days) may be another limiting factor concerning full display of the presumed effects of the medicine. A longer follow-up of the model allows for full-fledged development of the mechanisms underlying low-grade chronic inflammation and, consequently, CKD. A typical 8-week follow-up of the diabetic-and-obese rodent would mimic more closely the disease in humans [80,81,82]. However, a longer period of treatment, despite a higher chance of accurately noticing the beneficial effects, might lead to such an advanced degree of organ damage that it could taper the drug’s effectiveness. This aspect may constitute the fundament for further longer-duration studies. Furthermore, our results suggest that even a short-term administration of statin therapy may yield benefits, mostly in individuals affected by earlier stages of disease—diabetes and obesity, or metabolic syndrome.

A direct assessment of the precise effect size of Atorvastatin upon the severely metabolically impaired rodent required the presence of an additional work group model, diabetic-and-obese untreated rats. The reason we chose not to include this additional group was the peculiarly low 60-day survival rate of this model in a pilot study. In our paper, we compared the obese-and-diabetic rat treated with statin against the individual component diseases. It is expected that renal damage in the obese-and-diabetic (untreated) model is at least as severe as the more affected of the models separately displaying component pathologies—in this case, G-III. Similarly, the inflammatory profile in the forementioned composite group would demonstrate disturbances at least as severe as the worst independently ascertained group of comparison—again, G-III. Since we reported significant results by comparing the treated composite model against diabetic-and-obese rodents independently, the comparison against a composite untreated model should return results with benefits at least similar to the results in this paper.

Lastly, we employed a single statin representative of the class at a single weight-dependent dose. The most recent meta-analysis on the statin treatment effect upon renal function in CKD patients not on dialysis does not differentiate between statins for efficacy [83]. The current literature mentions that the dose of 20 mg/kg body weight yields a satisfactory balance between patient tolerability and adherence and cardiovascular protection. In many instances where the experimental design was focused upon the improvement of renal function in a STZ-induced model of diabetes, the Atorvastatin dose employed was within the lower range of values, never exceeding 40 mg/kg of body weight [81,82]. The diabetic nephropathy model benefits from a 10 mg/kg body weight dose, while the overlap of HFD rises slightly, up to 30 mg/kg of body weight [80]. Since our study used a relatively short follow-up period, we did not expect extensive renal damage. As a result, we decided upon a 20 mg/kg body weight dose, considering both the dual metabolic impairment from the model used and the research question of whether a short-term low-dose statin treatment would yield any improvement. Atorvastatin is a more hydrosoluble example of the class (accordingly, our outcomes are blood-derived parameters), and low-to-moderate doses should help avoid an increased risk for muscular symptoms. Nevertheless, parallel groups of different doses of Atorvastatin, or other statins at different equivalent doses, might exhibit varied results through different mechanisms for their anti-inflammatory and nephroprotective properties. Future studies on distinct doses and compounds are granted.

## 5. Conclusions

Obesity and diabetes mellitus are frequently encountered diseases that give rise separately to self-fueled destructive cycles. Currently, there are no guidelines directed at curbing overt inflammatory activity in these highly morbid diseases. Atorvastatin “wears many hats”, displaying a wide array of pleiotropic properties besides the wanted lipid profile improvement. Administration of statins in the current rodent model of dual metabolic impairment—glucose toxicity and high-fat diet—rescues, to a statistically significant extent, the renal function and part of the inflammatory profile. Most of the observations in our rodent model already have an equivalent counterpart effect in human research, but the conclusions are not readily translatable. Even though statins appear to effectively counteract the damage inflicted by a fat-rich diet in the uncontrolled diabetic model, this is not an endorsement for not adhering to the diet recommendations when prescribed lipid-lowering medication. Further research should focus on discovering the most efficient combined therapy to fully restore the inflammatory regulation ability.

## Figures and Tables

**Table 1:** Concise description of the rodent groups.

Group	Name	Experimental Protocol
G-I	Control (normal)	Rats fed with standard chow over whole study period
G-II	Obese (High-Fat Diet—HFD only)	Rats fed with HFD chow over whole study period
G-III	Diabetic (Diabetes-induced model only)	Streptozotocin-induced diabetes fed with standard chow
G-IV	Diabetic + Obese + Atorvastatin (Statin treated)	Streptozotocin-induced diabetes, HFD, and statin treated

**Table 2:** Macronutrient composition of diets prepared on demand. HFD, High-fat-content diet.

Chow (% by Weight)	Standard Lab Chow	Caloric Content	HFD + 2% Cholesterol	Caloric Content
Protein	23.7%	28.6%	18%	14.9%
Fat extract	5%	13.5%	31.7%	59.1%
Carbohydrate	48.4%	58.0%	31.3%	26%
Neutral detergent fiber	15.6%		14.7%	
Ash	7%		4.9%	
Energy density	3.34 kcal/gram		4.83 kcal/gram	

**Table 3:** A summary of relevant biochemical parameters at the two major moments of the study. CRP, C-reactive protein; HDL-Col, High-density lipoprotein fraction of cholesterol; LDL-Col, Low-density lipoprotein fraction of cholesterol. * denotes a statistically significant difference by Student’s *t*-test; independent samples at α = 0.05 between the marked group and the control G-I. ** denotes a statistically significant difference by bootstrap test with 5000 samples at α = 0.05 between the indicated group and control G-I. We did not assess statistical significance for lipid metabolism, since this was beyond this paper’s scope.

Time	Parameter/Group	G-I (Control)	G-II (Obese)	G-III (Diabetic Only)	G-IV (Statin Treated)
**Initial**	Glycemia (mg/dL)	89.9 (9.9)	86.3 (9.2)	84.7 (12.9)	98.8 (5.3) *
	Urea (mg/dL)	13.7 (2.0)	13.9 (2.8)	16.1 (3.0)	16.3 (3.1)
	Creatinine (mg/dL)	0.34 (0.09)	0.49 (0.16)	0.54 (0.12) *	0.42 (0.15)
	CRP (mg/dL)	2.0 (1.1, 3.0)	1.0 (0.7, 2.0)	1.6 (1.0, 1.8)	2.0 (1.0, 3.0)
**Final time point**	Glycemia (mg/dL)	84.1 (11.2)	99.6 (5.9) *	325.6 (43.1) *	277.4 (18.1) *
	Urea (mg/dL)	13.6 (2.4)	20.9 (1.6) *	67.6 (12.4) *	25.1 (2.9) *
	Creatinine (mg/dL)	0.26 (0.09)	0.76 (0.09) *	1.51 (0.18) *	0.89 (0.10) *
	CRP (mg/dL)	4.9 (0.8)	5.4 (1.5)	3.9 (1.3)	3.8 (1.0)
	Total Col (mg/dL)	176.7 (31.0)	250.7 (31.8)	260.0 (185.0, 262.0)	196.0 (189.0, 201.0)
	HDL-Col (mg/dL)	55.6 (6.8)	54.1 (7.7)	62 (5.5)	57.6 (8.6)
	LDL-Col (mg/dL)	52.9 (10.2)	50.6 (4.1)	39.2 (6.9)	92.4 (4.0)
	Triglycerides (mg/dL)	170 (164, 177)	170 (25)	193 (10)	194 (17)
	IL-1β (U/L)	68 (39)	4116 (427) *	3327 (623) *	4439 (795) *
	IL-6 (U/L)	66 (28)	3874 (2333) *	5689 (686) *	6817 (2564) *
	IL-17α (U/L)	180 (139)	830 (192) *	4002 (3856, 4752) **	3903 (2064, 4925) **
	TNFα (U/L)	129 (43)	1222 (324) *	3574 (545) *	2596 (942) *

**Table 4:** Histological proof for renal damage in the diabetic-and-obese statin-treated group G-IV.

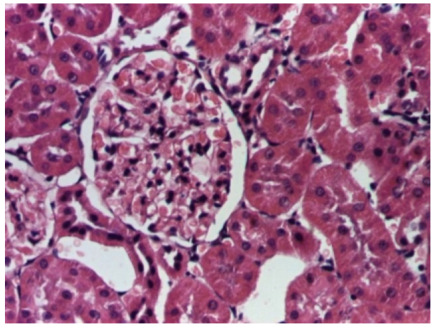	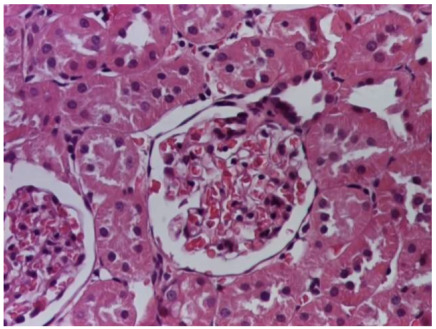
**4.1.** Hematoxylin and Eosin stain, 200× magnification. Normal aspect of the glomerulus, afferent arteriole, efferent arteriole, and normal renal tubules. Control group specimens.	**4.2.** Hematoxylin and Eosin stain, 200× magnification. Normal aspect of the juxtaglomerular apparatus. Control group specimens.
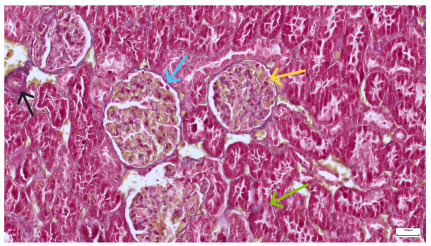	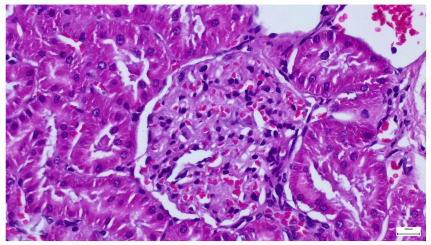
**4.3.** Trichrome Masson stain, 100× magnification, 200 microns scale segment. Arteriole thickening (black), glomerular basal membrane thickening (blue), mesangial cell expansion (yellow), proximal renal tubule basal membrane thickening (green). Group II obese High-Fat-Diet-fed specimens.	**4.4.** Hematoxylin and Eosin stain, 200× magnification, 100 microns scale segment. Glomerular congestion and mesangial cell expansion. Group II obese High-Fat-Diet-fed specimens.
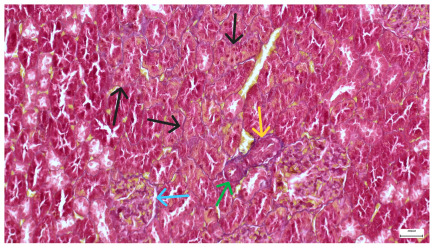	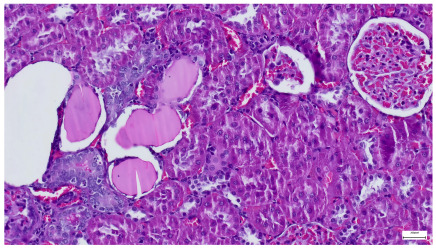
**4.5.** Trichrome Masson stain, 100× magnification, 200 microns scale segment. Arteriole wall thickening (green), unequal glomerular size and basal membrane thickening (blue), extensive proximal renal tubule basal membrane thickening (black), pronounced fibrosis in renal parenchyma (yellow). Group III diabetic and normal-diet-fed specimens.	**4.6.** Hematoxylin and Eosin stain, 100× magnification, 200 microns scale segment. Glomerular congestion, hyaline material present inside the lumen of renal tubules, flattened tubular walls. Group III diabetic and normal-diet-fed specimens.
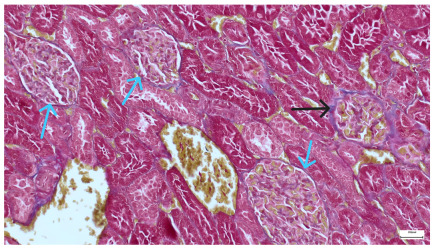	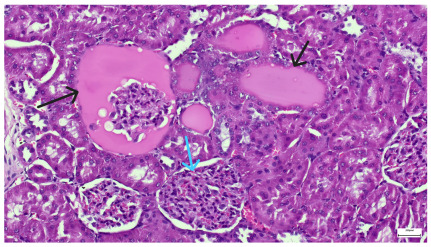
**4.7.** Trichrome Masson stain, magnification 100×, scale 200 microns. Renal corpuscle with relatively increased thickness of the glomerular basal membrane (black), renal corpuscular congestion, mesangial expansion, unequal glomerular dimensions (blue). Group IV diabetic-and-obese specimens.	**4.8.** Hematoxylin and Eosin stain, 100× magnification, scale 200 microns. Tubule “thyroidisation” (black) denoting the presence of eosinophilic material inside tubular lumen, the flattening of tubular wall cells as a consequence of glomerular partial or total sclerosis, significant hypercellular matrix environment in glomerulus (blue). Group IV diabetic-and-obese specimens.
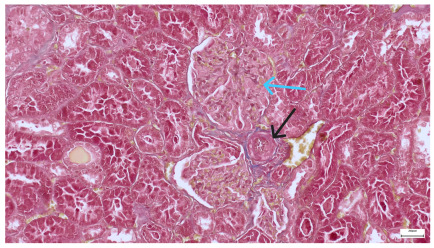	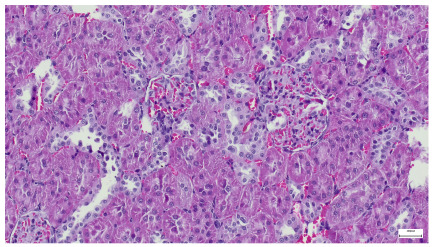
**4.9.** Trichrome Masson stain, magnification 100×, scale 200 microns. Arterial wall thickening is regarded as hyalinization (black), corpuscular congestion and start of segmental sclerosing (blue). Group IV diabetic-and-obese specimens.	**4.10.** Hematoxylin and Eosin stain, 100× magnification, scale 200 microns. Mesangial cell expansion and irregular glomerular dimensions. Group IV diabetic-and-obese specimens.

**Table 5:** Values for correlation (Pearson) or dependence (Kendall) between cytokines and urea or creatinine. Dependence was assessed on the whole (28) population. Correlation was limited to the (21) animals exposed to interventions. Boxes are colored red for lack of statistical significance and green for presence of statistical significance of the respective correlation/dependence at α = 0.05.

Predictor Variable	Coefficient Employed	IL-1β	IL-6	IL-17α	TNFα
**Urea**	Kendall τ	0.219	0.481	0.654	0.649
	Pearson *r*	−0.431	0.157	0.628	0.644
**Creatinine**	Kendall τ	0.271	0.436	0.667	0.689
	Pearson *r*	−0.426	0.056	0.627	0.649

## Data Availability

The original contributions presented in this study are included in the article/Supplementary Materials. Further inquiries can be directed to the corresponding authors.

## Supplementary Materials

The following supporting information can be downloaded at: https://www.mdpi.com/article/10.3390/life15081184/s1.

## Abbreviations

AMPK	Adenosine Monophosphate Kinase
AUC	Area under the Curve (for statistical methods)
DM	Diabetes Mellitus
HFD	High-Fat Diet
ICAM-1	Intercellular cell adhesion molecule-1
LGCI	Low-grade chronic inflammation
NF-κB	Nuclear Transcription Factor κB
NLRP3	NOD-like receptor family, pyrin-domain-containing inflammasome
NO	Nitrogen monoxide
OGTT	Oral glucose tolerance test
PECAM	Platelet endothelial adhesion molecule-1
RCT	Randomized controlled trial
STZ	Streptozotocin
TNFα	Tumoral necrosis factor α
TNFR	Tumoral necrosis factor α Receptor
T2DM	Type 2 diabetes mellitus
